# A Novel LSTM for Multivariate Time Series with Massive Missingness

**DOI:** 10.3390/s20102832

**Published:** 2020-05-16

**Authors:** Nazanin Fouladgar, Kary Främling

**Affiliations:** 1 Department of Computing Science, Umeå University, 901 87 Umeå, Sweden; kary.framling@umu.se; 2 School of Science and Technology, Aalto University, P.O. Box 15500, 00076 Aalto, Finland

**Keywords:** multivariate time series, regression, massive missingness, LSTM

## Abstract

Multivariate time series with missing data is ubiquitous when the streaming data is collected by sensors or any other recording instruments. For instance, the outdoor sensors gathering different meteorological variables may encounter low material sensitivity to specific situations, leading to incomplete information gathering. This is problematic in time series prediction with massive missingness and different missing rate of variables. Contribution addressing this problem on the regression task of meteorological datasets by employing Long Short-Term Memory (LSTM), capable of controlling the information flow with its memory unit, is still missing. In this paper, we propose a novel model called forward and backward variable-sensitive LSTM (FBVS-LSTM) consisting of two decay mechanisms and some informative data. The model inputs are mainly the missing indicator, time intervals of missingness in both *forward* and *backward* direction and *missing rate* of each variable. We employ this information to address the so-called missing not at random (MNAR) mechanism. Separately learning the features of each parameter, the model becomes adapted to deal with massive missingness. We conduct our experiment on three real-world datasets for the air pollution forecasting. The results demonstrate that our model performed well along with other LSTM-derivation models in terms of prediction accuracy.

## 1. Introduction

A great number of time series problems are stuck in Multivariate Time Series (MTS) on which multiple variables follow the interdependency between/within variables. Predicting future values of these variables by modeling previous observed sequences of values has been investigated widely among researchers for decades to make more accurate decisions [1,2,3,4,5]. For instance, meteorological data collected with different sensors is among multivariate time series problems involving different variables that change over time and accordingly predict future situations for target purposes. However, some difficulties in collecting such data range from faulty sensors to costly efforts of establishing them. This gives rise to the problem of missing data [6,7].

Multivariate time series with missing data is a challenge in different tasks, specifically in prediction. Since missingness causes bias in results, modeling any approach requires investigation of different types of missing data [8]. In general, one could categorize these data into three classes: *missing completely at random* (MCAR), *missing at random* (MAR) and *missing not at random* (MNAR). In the first class, the missing data lies independent of both the observed and unobserved variables, while in the second class, missingness falls dependent of the observed variables. In the case that there are some patterns of missingness but the observed variables cannot explain these patterns, the last class arises [9].

Various approaches have the potentiality of dealing with aforementioned missingness in MTS prediction. A straightforward policy is to discard the incomplete information and feed the complete information to traditional models like autoregressive moving average (ARMA) [10] or its generalized model, autoregressive integrated moving average (ARIMA) [11]. This policy could diminish the accuracy of prediction, specifically when it comes to MNAR missingness, due to the loss of rich information in the missing points of variables. Some researchers have applied other policies to impute the missing values with either simple statistics like mean or median [12] or more sophisticated statistics like polynomial interpolation [13], matrix factorization [14] and expectation maximization (EM) [15]. Furthermore, machine-learning algorithms like ANN [16], kNN [17] and decision tree [18] have been employed for the purpose of approximation and imputation of missing values. However, some of these approaches fall short in capturing the complex dynamisms of temporal dependencies within variables and some cannot deal with missingness when large amounts of data are lost or not collected.

Taking into account recurrent methods, the variants of Recurrent Neural Network (RNN) like Gated Recurrent Unit (GRU) [19,20,21] and Long Short-Term Memory (LSTM) [22,23,24] have demonstrated promising results in extracting temporal features and some could deal with huge missingness as well. It is notable that the approaches in [19,20,21,22] are among few works on addressing massive MNAR missingness in MTS (more than 70%). However, the main focus of these works have been on the classification task of medical domains. Exploring massive MNAR missingness in the regression task of other domains is still an open research area. More importantly, since not all variables follow the same missing rate in many applications, it is worth investigating the missing rate of each variable along with other missing information in the recurrent methods [21]. To the best of our knowledge, this has not yet been explored in an LSTM-based structure, having the power of controlling the information flow with its memory unit, jointly with other missing information.

This paper proposes a novel LSTM-based model called FBVS-LSTM to contribute to:

- dealing with massive MNAR missingness over three real-world meteorological and air quality MTS datasets [25,26,27], under the flag of regression task;
- exploring a new LSTM-based architecture, integrating jointly two decay mechanisms with the missing rate of each variable, to learn the missing pattern informatively;
- concluding that not all missing patterns provide informative data in the meteorological settings.

The rest of the paper is organized as follows. Section 2 considers related work in the domain of MTS with missingness. Section 3 gives details of the proposed method. We conduct our experiments on air pollution forecasting in Section 4. Finally, the conclusion and future works will be discussed in Section 5.

## 2. Related Works

Multivariate time series with missing values has been the main challenge in a great amount of literature for a long time [28]. Some works [29,30,31] put their attention on dealing with the MCAR missingness category mentioned in Section 1. In [29] a combination of three statistical models, namely vector autoregressive (VAR) [32], expectation and minimization (EM) and prediction error minimization (PEM) were applied to impute missing values. Normally, in an autoregressive model (AR) each sample is a linear combination of some previous observations with a stochastic term, while in a more generalized form, a vector autoregressive captures the linear interdependencies among multiple time series. The applied method identifies the series with different time lag, selects the best time lag and accordingly maximizes the likelihood of parameter estimation in the incomplete time series to impute missing values. Since the parameter estimation is computationally expensive in the EM process, Liu et al. [30] proposed sampling from Gaussian and gamma distributions as an alternative of sampling from conditional distribution in the EM process. In addition to [30], exploiting the precise model of MTS distribution for missingness imputation was discussed in [31]. The model exploits the power of generative adversarial networks to train the complete set and then apply the trained model for the imputation of the incomplete set. The imputation is done by finding the “closest” latent encoding of the missing value and then applying the generated samples of generator. Despite the effectiveness of these models [29,30,31], they did not prove the same performance in case of consecutive missingness. Addressing the consecutive gaps, Zhang et al. [33] proposed a deep-learning model consisting of encoder and decoder components. The model used the past and future information of latent factors in a bi-directional LSTM structure of encoder component. After applying a general attention mechanism to let the model focus on the data range of interest, uni-directional LSTM units accompanied by a fully connected layer were employed. Their main task was to reconstruct the learnt input data with missingness in a decoder component. Here, the assumption was that all variables follow the same missing rate, while in many applications the variables do not stand for the same characteristics and consequently the variables missing rate could differ.

Focusing on the MAR category of missingness in different applications [34,35], Feng et al. [34] manipulated the RNN structure to impute missingness in biomedical wearable recordings. Similar to our work, the model explored the latent features of missing values, using the forward and backward missing information. To impute missingness, while our model focuses on learning the past and future time lags in a jointly uni-directional manner, the current model puts attention on learning the history estimation as well as the feature estimation in a bi-directional manner. Shifting to the wind power prediction, Liu et al. [35] applied an EM-based estimation (like [29,30]) to impute missing values by estimating the mixture components of data distribution. Here, multiple imputation was performed to generate new samples and then let a Gaussian process regression signify how likely is a prediction, given the actual data.

Unlike the previous missingness categories, most of the literature on MNAR was mainly oriented towards health-related applications. Lipton et al. [36] worked on patient ICU records and modeled directly missing data by contributing a binary variable as well as other missing indicators. The variable basically indicated whether the data was measured or missed. Other missing indicators consisted of the mean and standard deviation of missing value, the relative times of the first and last recordings and also the frequency with which a variable switches from measured to missing or vice versa across adjacent sequence steps. All this information was attached to the input values and learnt in an LSTM-based structure. Similar to [36], Singh et al. [24] augmented the network input with some of the mentioned missing information, but in a bi-directional LSTM-based model. Moreover, the model was extended with some additional layers providing meaningful representations of missing data and more attention to the important spans of time series. Focusing again on an LSTM-based model, Kim et al. [22] presented a bio-inspired approach in terms of belief gate for the purpose of imputing missing data with either the last observation of each feature or its average. The work mainly focused on the impact of missingness forward and backward time intervals, interpreting as the extent of temporal belief one can trust on only the last observation at the current time. While this work considered the missing imputation within the stream, the work in [37] focused on the imputation and interpolation both across and within the stream, respectively. In general, all these models [22,24,36,37] exploited a fixed impact of time lags for the missingness imputations. Later, in [19,20], the authors addressed a dynamic impact of time lag under the flag of decay mechanism. The mechanism was imposed on both the input and latent factors in a GRU-based structure to explore more meaningful representations of missingness. Although in the latter works the missing rate of variables were huge (more than 70%) and different from each other, the model behaved the same for all variables. In [21], the missing rate of each variable was included in the inputs of GRU to reduce the harmful impact of variables with high missing rate on variables with low missing rate. Considering the fact that the mentioned literature of MNAR category was all analyzed over medical datasets, the work in [38] extended the analysis over the computer vision domain as well. The main idea was to reconstruct the missing values by not only the information of correlated observed features at the current time point but also other time points of the series. It is worth noting that all these models were articulated for the task of either classification or data reconstruction in MTS.

In this paper, we focus on the regression task of MTS in a different domain, mainly meteorological and air quality domain, with massive MNAR missingness. Moreover, different from [21], we investigate the potential capability of LSTM, by introducing a new structure. The structure mainly learns the joint information of two time lags under two decay mechanisms as well as the missing rate of each variable and its mask indicator.

## 3. Methods

### 3.1. LSTM

As a model of learning sequential data and capturing long term temporal dependencies, Long Short-Term Memory (LSTM) was first proposed by Hochreiter [39] in 1997. This model allows for constant error flow through self-connected units to impede from the gradient decay. In this regard, a memory cell along with three major gates construct the architecture of LSTM cell unit. The memory cell is mainly devised to keep or release the information by the contribution of three aforementioned gates. The gates are namely input gate $i_{t}$, forget gate $f_{t}$ and output gate $o_{t}$, by each the extent of information for passing forward is controlled. The following equations give more insight into the gates and process:

(1) $$f_{t}=\sigma \left(W_{f}x_{t}+U_{f}h_{t-1}+b_{f}\right)$$

(2) $$i_{t}=\sigma \left(W_{i}x_{t}+U_{i}h_{t-1}+b_{i}\right)$$

(3) $$o_{t}=\sigma \left(W_{o}x_{t}+U_{o}h_{t-1}+b_{o}\right)$$

(4) $$c_{t}=f_{t}\circ c_{t-1}+\left(i_{t}\circ \phi \left(W_{c}x_{t}+U_{c}h_{t-1}+b_{c}\right)\right)$$

(5) $$h_{t}=o_{t}\circ \phi \left(c_{t}\right)$$

In the equations above, σ stands for the sigmoid function, ϕ indicates the tanh function and ∘ stands for the element-wise multiplication. Furthermore, $x_{t}$ and $h_{t-1}$ are the inputs and hidden state at time *t* and t−1 respectively. The model parameters are also identified by *W*, *U* as weights and *b* as biases.

LSTM does not by itself contain any information of missingness to cope with multivariate time series with massive missingness. To make the model convenient for this purpose, we discuss two phases of modification. In the first phase, we propose an LSTM with two decay mechanisms, namely forward and backward LSTM (FB-LSTM). This model contains the *mask indicator* and *time intervals* as two important information of missing pattern. In the second phase, we extend FB-LSTM to a variable-sensitive version, namely FBVS-LSTM. Here, we incorporate the missing rate of each variable to deal with massive missingness problem. In the following, the aforementioned models are articulated in Section 3.2 and Section 3.3 respectively.

### 3.2. FB-LSTM

To deal with MNAR category of missing data, we modify LSTM with two decay mechanisms, namely forward and backward LSTM (FB-LSTM). The decay mechanisms are devised to reinforce the imputation of missing values more accurately. Similar to [19], the missing data is formulated with a missing indicator $M={\left\{m_{1},m_{2},\ldots ,m_{T}\right\}}^{T}\in R^{D\times T}$ for each observation $x_{t}^{d}$ in the time series $X={\left\{x_{1},x_{2},\ldots ,x_{T}\right\}}^{T}\in R^{D\times T}$ where d∈{1,…,D} and t∈{1,…,T} denote the variable and time of observation, respectively. In this regard, the observation, $x_{t}^{d}$ is interpreted as the *t*-th observation of the variable *d*. Following the formulations, the missing indicator *m* at time stamp *t* of the variable *d*, $m_{t}^{d}$, is regarded as a binary mask as below:

(6) $$\;m_{t}^{d}=\{\begin{array}{cc} 1, & \mathrm{if}\;x_{t}^{d}\;\mathrm{is}\;\mathrm{observed}\\ 0, & \mathrm{otherwise}. \end{array}\;$$

In addition to such information, suppose that in the process of meteorological data collection, the sensors of capturing air pollution have low sensitivity to the high pollution. Therefore, the data gathering would drop in such case of pollution. The process will be back on the track of data collection when the level of pollution decreases. In this context, considering the duration of missingness is critical to be explored as an informative missing pattern. To address this, in each time stamp, the last observation of each variable from current missing data, as well as the first observation after that, are calculated. The former follows the same formulation as [19], incorporating our first decay mechanism and the latter is formulated in this work, incorporating our second decay mechanism. While the first mechanism contributes in the decreasing impact of backward time interval, the second mechanism does the same in the forward time interval. In the following, similar to the definition in [19], the time interval between the last observation and current missing data for each variable *d* at time stamp *t* is defined as $\delta_{t}^{1d}$ in a set of $\delta^{1}={\left\{{\delta_{1}}^{1},{\delta_{2}}^{1},\ldots ,{\delta_{T}}^{1}\right\}}^{T}\in R^{D\times T}$:

(7) $$\;\delta_{t}^{1d}=\{\begin{array}{cc} s_{t}-s_{t-1}+\delta_{t-1}^{1d}, & t>1,m_{t-1}^{d}=0\\ s_{t}-s_{t-1}, & t>1,m_{t-1}^{d}=1\\ 0, & t=1 \end{array}\;$$

In the equation above, $s_{t}$ is the time stamps when the *t*-th observation is recorded.

In this paper, we define $\delta^{2}={\left\{{\delta_{1}}^{2},{\delta_{2}}^{2},\ldots ,{\delta_{T}}^{2}\right\}}^{T}\in R^{D\times T}$ as the set of time intervals between the current value and first observation after that. Therefore, $\delta_{t}^{2d}$ indicates the forward time interval for each variable *d* at time *t*:

(8) $$\;\delta_{t}^{2d}=\{\begin{array}{cc} s_{t+1}-s_{t}+\delta_{t+1}^{2d}, & t>1,m_{t+1}^{d}=0\\ s_{t+1}-s_{t}, & t>1,m_{t+1}^{d}=1\\ 0, & t=1 \end{array}\;$$

Although the two defined sets of time intervals could reflect the useful information influencing the missing data, their impact on missingness decrease as the time intervals increase. Therefore, two decay rates are defined, implying for the first and second decay mechanisms. The rates mainly control the impacts of time intervals over time and contribute in the process of learning with other model parameters in LSTM. The first decay rate, $\gamma^{1}$, is formulated as [19] and the second one, $\gamma^{2}$, is introduced in this work:

(9) $$\gamma^{1}=exp\left\{-max\left(0,{W_{\gamma }}^{1}\delta^{1}+{b_{\gamma }}^{1}\right)\right\}$$

(10) $$\gamma^{2}=exp\left\{-max\left(0,{W_{\gamma }}^{2}\delta^{2}+{b_{\gamma }}^{2}\right)\right\}$$

where ${W_{\gamma }}^{1}$, ${W_{\gamma }}^{2}$, ${b_{\gamma }}^{1}$ and ${b_{\gamma }}^{2}$ are the model parameters. It should be mentioned that the decay rates range between 0 and 1.

To learn the parameters, the decay rates are imposed jointly to the input and hidden features of LSTM to capture the missing pattern informatively. This process constructs the main structure of LSTM with two decay mechanisms (FB-LSTM).

In FB-LSTM, the missing data is imputed with the values either close to the mean of the variable or close to the last/first observation of the variable. This basically implies the interpretation that the smaller the time intervals are, the closer the missing data is to the last/first observation of the variable. Moreover, this data is closer to the mean of the variable if the time intervals are larger. To formulate this, we denote ${\tilde{x}}^{d}$ as the mean of variable *d*. The last and first observations of variable *d* are also indicated by $x_{t^{\prime }}^{d}$ and $x_{t^{\prime \prime }}^{d}$, respectively. The Equation (11) shows the decay process over the input mathematically:

(11) $${\hat{x}}_{t}^{d}=m_{t}^{d}x_{t}^{d}+\left(1-m_{t}^{d}\right)\left[\left(\gamma_{xt}^{1d}x_{t^{\prime }}^{d}+\;\gamma_{xt}^{2d}x_{t^{\prime \prime }}^{d}\right)+\;\left(\left(1-\gamma_{xt}^{1d}\right)\left(1-\gamma_{xt}^{2d}\right){\tilde{x}}^{d}\right)\right]$$

where $\gamma_{xt}^{1d}$ and $\gamma_{xt}^{2d}$ represent the input decay rates of the first and second decay mechanisms, respectively.

In addition to imposing the two decay mechanisms on the input, we apply the same mechanisms on the hidden state to facilitate exploring the rich information of missing data in the latent space. More clearly, the two decay rates make their influence simultaneously on the previous hidden state as Equation (12):

(12) $${\hat{h}}_{t-1}=\gamma_{ht}^{1}\gamma_{ht}^{2}⊙h_{t-1}$$

in which $\gamma_{ht}^{1}$ and $\gamma_{ht}^{2}$ represent the hidden state decay rates.

Then, the obtained input and hidden state from Equations (11) and (12) directly incorporate in LSTM gates, described in Section 3.1, to construct the structure of FB-LSTM. Furthermore, the masking indicator is added to all the three gates to let the model learn from the missingness directly. The following equations illustrate the FB-LSTM functionality over the cell memory and all the gates:

(13) $$f_{t}=\sigma \left(W_{f}{\hat{x}}_{t}+U_{f}{\hat{h}}_{t-1}+V_{f}m_{t}+b_{f}\right)$$

(14) $$i_{t}=\sigma \left(W_{i}{\hat{x}}_{t}+U_{i}{\hat{h}}_{t-1}+V_{i}m_{t}+b_{i}\right)$$

(15) $$o_{t}=\sigma \left(W_{o}{\hat{x}}_{t}+U_{o}{\hat{h}}_{t-1}+V_{o}m_{t}+b_{o}\right)$$

(16) $$c_{t}=f_{t}\circ c_{t-1}+\left(i_{t}\circ \phi \left(W_{c}{\hat{x}}_{t}+U_{c}{\hat{h}}_{t-1}+V_{c}m_{t}+b_{c}\right)\right)$$

(17) $$h_{t}=o_{t}\circ \phi \left(c_{t}\right)$$

in which $V_{f}$, $V_{i}$, $V_{o}$ and $V_{c}$ are the added parameters in FB-LSTM.

### 3.3. FBVS-LSTM

In many MTS, each variable follows its own characteristics, implying different frequency in the case of missingness. This characteristic is critical to explore, specifically when the variables with high missing frequency negatively influence those with low missing frequency. Since FB-LSTM only considers the missing indicator and the time intervals of missingness for each variable, we extend this model to a variable-sensitive version, namely forward and backward variable-sensitive LSTM (FBVS-LSTM). In the following, we explain how this model works.

First, the missing rate of each variable *d*, $\mu^{d}$, over all time steps *t* is calculated by the mask indicator $m_{t}^{d}$. We formulate this rate similar to [21]:

(18) $$\mu^{d}=1-\frac{1}{T}\sum_{t=1}^{T}m_{t}^{d}$$

where $\mu^{d}$ ranges between 0 and 1.

Then, to make the model adapted to the missing rate of each variable independently, there is a possibility of contributing $\mu^{d}$ in the learning process of previously articulated model (FB-LSTM) and accordingly constructing FBVS-LSTM model. The advantage of this contribution is to make the model sensitive to the variables with low missingness. However, subsuming $\mu^{d}$ directly in FB-LSTM, the model cannot discern the particular missingness feature of variables for those with close missing frequency. This is notable, specifically when $\mu^{d}$ is close to 1 in case of massive missingness. Therefore, the missing rate of each variable is decayed within a negative exponential function to construct a missing factor β. Later, this factor directly takes part in the learning process in FBVS-LSTM. The formulation of β is similar to [21] as below:

(19) $$\beta =exp\{-max\left(0,W_{\beta }\mu +b_{\beta }\right)\}$$

where $W_{\beta }$ is a vector as size as the transpose of missing rates vector μ, and $b_{\beta }$ is also a vector as size as μ. $W_{\beta }$ stands for the weights and $b_{\beta }$ indicates the bias, integrating with μ.

**Figure 1:**
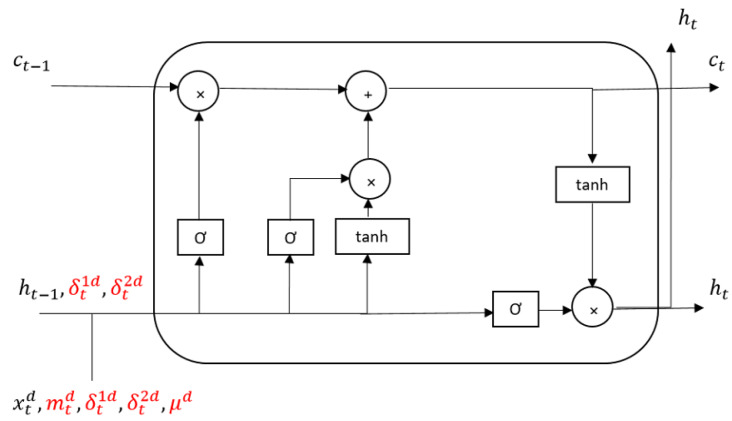
FBVS-LSTM unit.

Incorporating the missing factor β in FB-LSTM, the gates are rectified as the equations below and construct our final model as FBVS-LSTM:

(20) $$f_{t}=\sigma \left(W_{f}{\hat{x}}_{t}+U_{f}{\hat{h}}_{t-1}+V_{f}m_{t}+P_{f}\beta +b_{f}\right)$$

(21) $$i_{t}=\sigma \left(W_{i}{\hat{x}}_{t}+U_{i}{\hat{h}}_{t-1}+V_{i}m_{t}+P_{i}\beta +b_{i}\right)$$

(22) $$o_{t}=\sigma \left(W_{o}{\hat{x}}_{t}+U_{o}{\hat{h}}_{t-1}+V_{o}m_{t}+P_{o}\beta +b_{o}\right)$$

(23) $$c_{t}=f_{t}\circ c_{t-1}+\left(i_{t}\circ \phi \left(W_{c}{\hat{x}}_{t}+U_{c}{\hat{h}}_{t-1}+V_{c}m_{t}+P_{c}\beta +b_{c}\right)\right)$$

(24) $$h_{t}=o_{t}\circ \phi \left(c_{t}\right)$$

in which *W*, *U*, and *V* are the parameters of model. These parameters are regarded as vectors instead of matrixes. This could accelerate the learning process in FBVS-LSTM with less parameter computation than FB-LSTM. Another parameter of FBVS-LSTM compared with FB-LSTM is the vector *P*, responsible for learning the missing factor. Considering massive reduction of computation in FBVS-LSTM, this vector does not overload more than the entire computations in FB-LSTM. The cell structure of FBVS-LSTM is depicted in Figure 1.

## 4. Experiments

### 4.1. Dataset Description and Preprocessing

The proposed method is performed on three real datasets, namely Beijing PM2.5 [25], Italy Air Quality [26] and Beijing Multi-Site Air Quality [27], collections of hourly meteorological and air quality data. The first dataset consists of 8 main attributes, namely *PM2.5 concentration, dew point, temperature, pressure, wind direction, cumulated wind speed, cumulated hours of snow* and *cumulated hours of rain*. The values have been gathered for the period of 1 January 2010 to 31 December 2014. This period contains 43,824 hourly instances quite big data. To reduce the time complexity, we select only one year of data over the period of 1 January 2010 till 31 December 2010, consisting of 8760 samples. The second dataset encompasses the average responses of 5 metal oxide chemical sensors in one of the polluted areas in an Italian city. The attributes are mainly PT08.S1 (tin oxide), PT08.S2 (titania), PT08.S3 (tungsten oxide), PT08.S4 (tungsten oxide) and PT08.S5 (indium oxide), nominally stand for CO, NMHC, NOx, NO2 and O3. Considering 8760 out of 9358 samples, we choose again only one year of data, from 11 March 2004 to 11 March 2005. Moreover, employing the data collected from multiple sites in Beijing, the third dataset stands for 6 features in Guanyuan site, namely PM2.5, PM10, SO2, NO2, CO and O3 concentrations. Similar to two previous datasets, we consider a subset of data to reduce time complexity. Here, 7346 out of 35,065 instances from the period of 1 March 2013 till the third data of 1 January 2014 are opted.

We reshape the samples and generate a multivariate time series of 24 h within 8, 5 and 6 variables, standing for each of our three datasets, respectively. These samples are required to feed into our models, discussed further in Section 4.3, for the purpose of short-term (next-hour) prediction. In order to explore the imputation impact of variables with massive missingness on the prediction task, first we forecast a variable with a high missing rate in the first dataset and later we predict variables with low missing rate in the second and third datasets, respectively. In the first dataset, a one-step-ahead prediction of PM2.5 concentration over the last 24 h is performed. In the cases of the second and third datasets, we focus on the next-hour prediction of PT08.S1 (tin oxide), saying PT08.S1 (CO), and O3, respectively over the last 24 h. It should be mentioned that we drop the first 24 h in the first dataset, making our data uniform when generating the MNAR missingness.

To synthetically generate MNAR missingness, we make usage of each feature median [40] in our datasets. Since we aim to achieve high rate of missingness, we consider different formulations, yet the same logic, in each dataset. In Beijin PM2.5, we subtract 0.6×median of each feature from its median. In fact, two groups of values are defined given this subtraction. One group has the higher values than the subtraction value and the other group has the lower values. The first group is represented as missingness while the second group keeps the observed values. The following formulation indicates this process for all features:

(25) $$\;x_{t}^{d}=\{\begin{array}{cc} observed, & \mathrm{if}\;x_{t}^{d}<median\left(x^{d}\right)-0.6*median\left(x^{d}\right)\\ missing, & \mathrm{otherwise}. \end{array}\;$$

However, by applying Equation (25) on the *PM2.5 concentration* feature, all values of this feature are lost. Therefore, we apply only the median of this variable as the decision point of missingness. It should be pointed out that we refer to this feature as PM2.5 for simplicity. Equation (26) shows this formulation:

(26) $$\;x_{t}^{PM2.5}=\{\begin{array}{cc} observed, & \mathrm{if}\;x_{t}^{PM2.5}<median\left(x^{PM2.5}\right)\\ missing, & \mathrm{otherwise}. \end{array}\;$$

With the same policy, we formulate missingness in each feature of the second and third datasets. Here, we discriminate PT08.S3 (tungsten oxide), saying PT08.S3(NOx) and SO2 in these two datasets, applying different formulations to provide massive missingness. Equations (27) and (28) indicate the formulations of MNAR missingness generation over all features of the second and third datasets respectively, excluding PT08.S3(NOx) and SO2 features.

(27) $$\;x_{t}^{d}=\{\begin{array}{cc} observed, & \mathrm{if}\;x_{t}^{d}<median\left(x^{d}\right)+0.1*median\left(x^{d}\right)\\ missing, & \mathrm{otherwise}. \end{array}\;$$

(28) $$\;x_{t}^{d}=\{\begin{array}{cc} observed, & \mathrm{if}\;x_{t}^{d}<median\left(x^{d}\right)+0.5*median\left(x^{d}\right)\\ missing, & \mathrm{otherwise}. \end{array}\;$$

Additionally, we generate missing data over PT08.S3(NOx), and SO2 attributes, employing the following equations:

(29) $$\;x_{t}^{NOx}=\{\begin{array}{cc} observed, & \mathrm{if}\;x_{t}^{NOx}<median\left(x^{NOx}\right)-0.3*median\left(x^{NOx}\right)\\ missing, & \mathrm{otherwise}. \end{array}\;$$

(30) $$\;x_{t}^{SO2}=\{\begin{array}{cc} observed, & \mathrm{if}\;x_{t}^{SO2}<median\left(x^{SO2}\right)-0.6*median\left(x^{SO2}\right)\\ missing, & \mathrm{otherwise}. \end{array}\;$$

It should be mentioned that we used “NOx” as an abbreviation for “PT08.S3(NOx)” variable in Equation (29).

**Figure 2:**
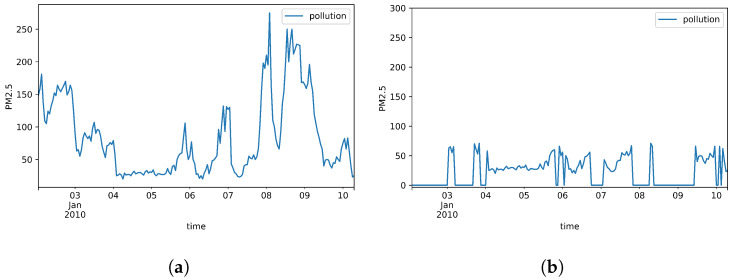
PM2.5 concentration over 200 samples in Beijin PM2.5. (**a**) Actual data; (**b**) Generated missing data.

**Figure 3:**
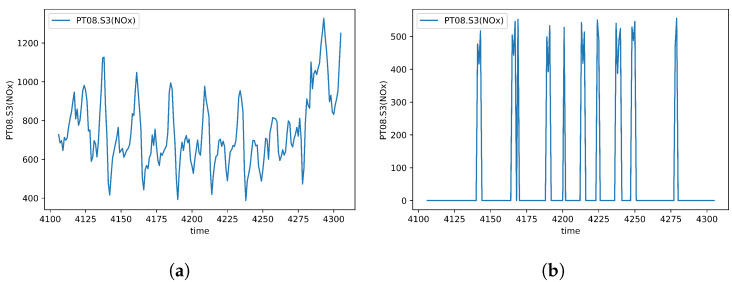
NOx over 200 samples in Italy Air Quality. (**a**) Actual data; (**b**) Generated missing data.

**Figure 4:**
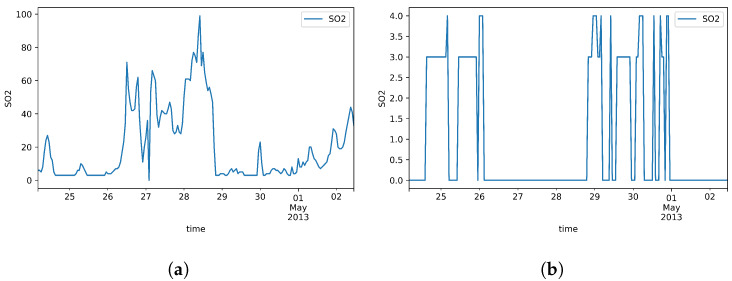
SO2 over 200 samples in Beijing Multi-Site Air-Quality. (**a**) Actual data; (**b**) Generated missing data.

After applying the formulations above, the approximate missing rate of all datasets features are calculated and presented in Table 1. In addition, to visually compare the actual values of features and their generated missingness, Figure 2, Figure 3 and Figure 4 are depicted over 200 samples standing for PM2.5, the attribute of first dataset, as well as NOx and SO2, the attributes of second and third datasets, respectively.

### 4.2. Metric

To measure the performance of models in the regression task of all datasets, we consider the mean squared error (MSE), mathematically represented as follows:

(31) $$MSE=\frac{1}{n}\sum_{i=1}^{n}{\left(y_{i}-{\hat{y}}_{i}\right)}^{2}$$

### 4.3. Evaluation and Results

In this section, we evaluate the results of proposed method along with five variations of LSTM as assessment models over all datasets. The assessment models mainly consist of LSTM with zero imputation, LSTM with mean imputation, LSTM with first decay mechanism, LSTM with second decay mechanism and variable-sensitive LSTM equipped with the first decay mechanism. We refer these models as LSTM-0, LSTM-mean, B-LSTM, F-LSTM and BVS-LSTM, respectively. The first model imputes missing data with zero and the second model imputes with the mean value of each variable in all time stamps. While the third model imputes missingness considering the forward time interval, $\delta^{1}$, the fourth model follows the same procedure considering the backward time interval, $\delta^{2}$. Finally, the last model employs both $\delta^{1}$ and μ, missing rate of each variable, for imputation. Applying any of these models, the mask indicator is also fed into the model to contribute in identifying the missing pattern directly.

It is worth pointing out that time series data of each dataset are normalized before feeding into the network to scale all features in the same range. This process is accomplished by max-min normalization and ranges data between 0 and 1 as follows:

(32) $$x_{norm}=\frac{x-x_{min}}{x_{max}-x_{min}}$$

**Figure 5:**
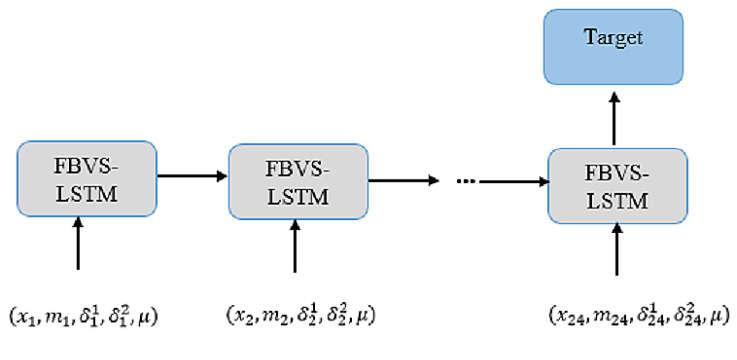
The general architecture of FBVS-LSTM units.

The normalized time series are then appraised with 5-fold cross-validation on the assessment models as well as the proposed model. In each fold, the models of each dataset are separately trained within 24 LSTM units implying for 24 h training data. More clearly, each unit stands for one hour of training data. Figure 5 illustrates the whole architecture of the proposed model with the 24 units.

Moreover, the training process of each fold accomplishes a 30-epoch run for each model. As mentioned before, the output is one-step-ahead prediction of appropriate variable in each dataset, indicating the next-hour prediction from the current time. Therefore, we tune the number of outputs to 1 for all datasets. Since there are 8, 5 and 6 features in the first, second and third datasets respectively, the input size of each dataset is adjusted correspondingly. Furthermore, the learning rate is initiated with 0.01 and the optimizer to train each model is considered to be stochastic gradient descent. The parameter settings of all datasets are shown in Table 2.

Table 3 shows the results of comparison between each assessment model and the proposed method in three datasets. The results are represented in terms of training and test sets MSE errors as well as their standard deviation. It is worth pointing out that the errors imply for the average loss errors of 5-fold cross-validation in each model of each dataset. Considering the results of first dataset, we could verify that FBVS-LSTM performs rather similar to BVS-LSTM during the training process with a slight error difference. This reveals the fact that the forward time interval does not provide much more effective information than the integration of backward time interval and missing rate provides. This claim is also asserted by comparing the test errors of these two models. In this case, FBVS-LSTM performs again tightly in line with BVS-LSTM. Here as well as the training set, F-LSTM has the highest amount of errors among other models and this could strengthen the truth of our previous argument that the latent factors of future are not strong representations of missing pattern in this dataset. This is mainly due to the rather non-discriminative future pattern in a local window. Comparing other assessment models, LSTM-0 and LSTM-mean show quite similar performance to each other during the training and testing process, while B-LSTM outperforms these two models. However, statistically all these three models as well as BVS-LSTM perform quite similar to FBVS-LSTM. In general, closing the errors to zero proves the robustness of all these LSTM-based models and indicates that the variables with high rate of missingness influence those with low rate less. This is because the models with the power of their gates and cell memory could regulate the extent of missingness imputation in a local window within the variables. This is true even if there is no information of missingness. In case of such information, the models also generate an estimation of each feature for regulation. Moreover, by separately learning the information of missing rate, the variables with high missingness could less influence those with low missingness.

The results extracted from the second dataset in terms of the training and test errors indicate that FBVS-LSTM outperforms other models with the minimum error difference around 0.01 over BVS-LSTM and the maximum error difference around 0.8 over LSTM-0. This verifies that all four information of missingness employed in FBVS-LSTM influenced the imputation process more accurate than other models, specifically LSTM-mean and LSTM-0. These two models performed more weakly relative to other models. Considering the fact that this dataset encompasses high variations (see Figure 3), LSTM-mean and LSTM-0 encounter with the lack of additional information to extract latent factors. Yet, relying on the forget gate in LSTM-0 as well as the mean of variables in LSTM-mean provide promising results. Among other models, here F-LSTM also shows promising results and this is mainly due to equipping with additional information when it comes to a dataset with high variation. The same argument stands for B-LSTM as well. In fact, F-LSTM and B-LSTM models accompanied with BVS-LSTM show quite similar performance to our proposed model in this dataset.

In the third dataset, the presented model performs tightly in line with B-LSTM in both training and test set errors. Comparing FBVS-LSTM with BVS-LSTM, B-LSTM and F-LSTM, it proves that forward time intervals in missing data does not provide informative representation of missingness. The claim is similar to the results obtained from the first dataset. Focusing on LSTM-mean and LSTM-0, the former presents more accurate imputation due to the input inclination toward the mean of variable. In future works, we could test and analyze whether the same situation exists for other statistical attributes of variable. In general, all models except F-LSTM shows promising results.

**Figure 6:**
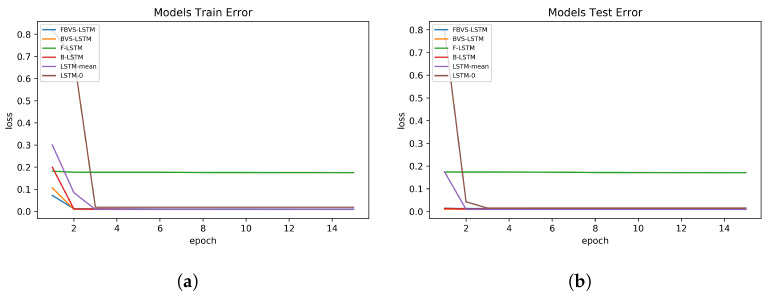
Models performance in Beijing PM2.5 dataset. (**a**) Training set errors; (**b**) Test set errors.

**Figure 7:**
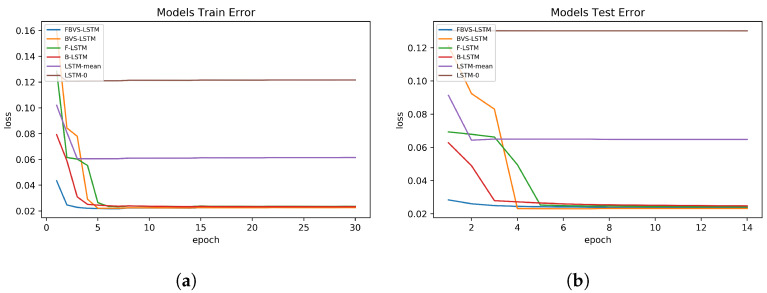
Models performance in Italy Air Quality dataset. (**a**)Training set errors; (**b**) Test set errors.

**Figure 8:**
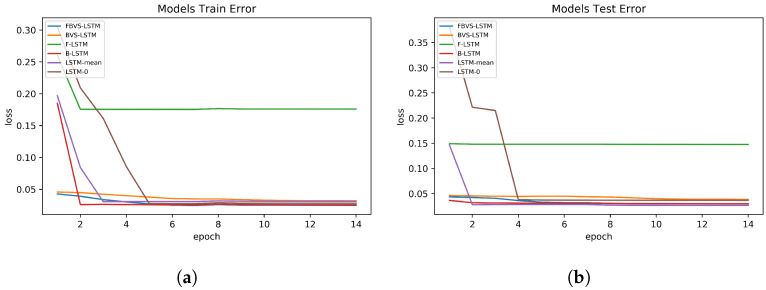
Models performance in Beijing Multi-Site Air-Quality dataset. (**a**) Training set errors; (**b**) Test set errors.

**Figure 9:**
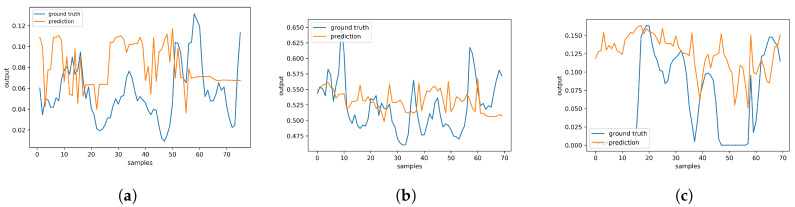
Prediction and ground truth outputs over 70 test samples. (**a**) PM2.5 in Beijing PM2.5 dataset; (**b**) PT08.S1(CO) in Italy Air Quality dataset; (**c**) O3 in Beijing Multi-Site Air-Quality.

Visually exploring how the models of each dataset perform in the training and test sets, Figure 6, Figure 7 and Figure 8 are depicted in 15 epochs. Each epoch illustrates the average errors of the same epoch within 5-fold cross-validation. As shown in Figure 6, in the first dataset, all models except F-LSTM follow a decreasing pattern until they reach constant error levels. As mentioned before, F-LSTM could not provide informative representation of missingness in this dataset. Therefore, this method pursues a constant trend within all epochs. The same analysis is valid in the third dataset as well (Figure 8), except the decreasing trend of F-LSTM within the primitive epochs in the training set. Among other models in the first and third datasets, although FBVS-LSTM learns the missing pattern with slightly lower loss error than LSTM-0, LSTM-mean and BVS-LSTM from the primitive stages, these models also perform well with loss errors close to zero. This argument stands for both the training and test sets in both datasets. In case of B-LSTM, the model produces higher errors than FBVS-LSTM in the training sets of first and third datasets (Figure 6a and Figure 8a). However, it shows similar performance to FBVS-LSTM in the test sets of first dataset (Figure 6b) and a lower loss start in the test set of third dataset (Figure 8b). Considering model performance in the second dataset, LSTM-0 and LSTM-mean encounter higher errors in the training and test sets from the primitive epochs as expected (Figure 7a,b). Other models as well as the proposed model follow a decreasing pattern in both training and test sets, yet lower loss in the beginning refers to FBVS-LSTM. In general, we could conclude that most of LSTM-based models perform well dealing with massive MNAR missingness in the regression task of meteorological settings. To realize the accuracy of proposed model visually, the prediction and ground truth of PM2.5, PT08.S1(CO) and O3 variables over 70 test data samples are depicted for their corresponding dataset in Figure 9.

### 4.4. Statistical Analysis

To acknowledge the achieved results of Section 4.3, a statistical analysis has been employed to compare the proposed algorithm (FBVS-LSTM) with the introduced assessment models in each of the three datasets. In this paper, we conduct the t-test as one of the most commonly applied analysis. Two hypotheses, H0 and H1, are defined as below:

- **H0**: *The proposed method performed similarly w.r.t. other assessment models.*
- **H1**: *The proposed method performed differently w.r.t. other assessment models.*

Here, we consider 30 tests. In addition, the degrees of freedom and alpha are adjusted to 58 and 0.05, respectively. Therefore, there is 95% confidence that the conclusion of test is valid. Table 4 shows the result of *t*-test in terms of *t*-value and *p*-value in each of the applied datasets and their models. It can be seen that in the first dataset, F-LSTM has the *p*-value equals to 0.0001, much less than 0.05, indicating the acceptance of rejecting the null hypothesis. With the same logic, LSTM-0 and LSTM-mean reject the null hypothesis in the second dataset with p-values equal to 0.0002 and 0.0005, respectively. In the third dataset, B-LSTM model accepts the null hypothesis with the *p*-value equals to 0.71, much higher than 0.05. All other models of this dataset reject H0 and accept H1.

## 5. Conclusions and Future Works

This paper was conducted to address the massive MNAR missingness on the regression task of meteorological multivariate time series. We proposed a novel LSTM-based model, FBVS-LSTM, consisting of four effective pieces of information as the augmentation of model input. The information included the missing indicator, two time intervals of missingness in forward and backward direction and missing rate of each variable. Experiments were conducted on Beijing PM2.5, Italy Air Quality and Beijing Multi-Site Air Quality datasets, filtering out around one year of data of all datasets for short-term prediction. The results proved promising performance of the proposed model along with some LSTM-based derivative methods. More importantly, we concluded that not all missing patterns provide meaningful representation of missingness. Future study aims to replicate the experiments in a health-related domain, mainly a daily stress-monitoring dataset collected from different sensors. Additionally, we will explore how the deeper layers of LSTM could affect the performance of model. Finally, further evaluations with other deep-learning models like GRU will be performed to investigate which gates have the most influential role to deal with missingness.

## Figures and Tables

**Table 1:** Missing rate of datasets features.

Dataset	Features	Missing Rate
Beijing PM2.5	PM2.5	75%
	dew	52%
	temperature	66%
	pressure	49%
	wind direction	89%
	wind speed	67%
	snow	1%
	rain	5%
Italy Air Quality	PT08.S1(CO)	34%
	PT08.S2(NMHC)	38%
	PT08.S3(NOx)	88%
	PT08.S4(NO2)	32%
	PT08.S5(O3)	45%
Beijing Multi-Site Air-Quality	PM2.5	34%
	PM10	28%
	SO2	81%
	NO2	22%
	CO	36%
	O3	38%

**Table 2:** Parameter settings.

Datasets	Parameters				
	Epoch Number	Learning Rate	Hidden Layers	Features (Input Size)	Output Size
Beijing PM2.5	30	0.01	24	8	1
Italy Air Quality	30	0.01	24	5	1
Beijing Multi-Site Air-Quality	30	0.01	24	6	1

**Table 3:** All models performance in each dataset.

Dataset	Model	MSE ± STD	
		Train Error	Test Error
Beijin PM2.5	LSTM-0	0.021 ± 0.020	0.016 ± 0.009
	LSTM-mean	0.021 ± 0.016	0.015 ± 0.011
	B-LSTM	0.016 ± 0.008	0.010 ± 0.004
	F-LSTM	0.180 ± 0.324	0.172 ± 0.323
	BVS-LSTM	0.013 ± 0.006	0.010 ± 0.004
	FBVS-LSTM	0.012 ± 0.004	0.011 ± 0.005
Italy Air Quality	LSTM-0	0.122 ± 0.123	0.130 ± 0.142
	LSTM-mean	0.063 ± 0.078	0.066 ± 0.082
	B-LSTM	0.027 ± 0.003	0.027 ± 0.009
	F-LSTM	0.030 ± 0.007	0.029 ± 0.015
	BVS-LSTM	0.031 ± 0.011	0.031 ± 0.013
	FBVS-LSTM	0.023 ± 0.002	0.024 ± 0.006
Beijing Multi-Site Air-Quality	LSTM-0	0.049 ± 0.029	0.06± 0.032
	LSTM-mean	0.038 ± 0.015	0.03 ± 0.023
	B-LSTM	0.031 ± 0.011	0.03 ± 0.016
	F-LSTM	0.179 ± 0.3	0.148 ± 0.25
	BVS-LSTM	0.034 ± 0.008	0.040 ± 0.024
	FBVS-LSTM	0.026 ± 0.019	0.031 ± 0.002

**Table 4:** Statistical analysis with *t*-test.

Dataset	Model	FBVS-LSTM	
		*t*-Value	*p*-Value
Beijin PM2.5	LSTM-0	−1.62	0.11
	LSTM-mean	−0.87	0.39
	B-LSTM	−0.5	0.61
	F-LSTM	−78.23	0.0001
	BVS-LSTM	−0.02	0.98
Italy Air Quality	LSTM-0	−135.68	0.0002
	LSTM-mean	−24.17	0.0005
	B-LSTM	−1.58	0.12
	F-LSTM	−1.87	0.06
	BVS-LSTM	−1.51	0.13
Beijing Multi-Site Air-Quality	LSTM-0	−15.07	0.0001
	LSTM-mean	−868.89	0.0004
	B-LSTM	−0.37	0.71
	F-LSTM	−52.92	0.0008
	BVS-LSTM	−6.00	0.0001
